# Treatment with hyperimmune equine immunoglobulin or immunoglobulin fragments completely protects rodents from Ebola virus infection

**DOI:** 10.1038/srep24179

**Published:** 2016-04-12

**Authors:** Xuexing Zheng, Gary Wong, Yongkun Zhao, Hualei Wang, Shihua He, Yuhai Bi, Weijin Chen, Hongli Jin, Weiwei Gai, Di Chu, Zengguo Cao, Chong Wang, Quanshui Fan, Hang Chi, Yuwei Gao, Tiecheng Wang, Na Feng, Feihu Yan, Geng Huang, Ying Zheng, Nan Li, Yuetao Li, Jun Qian, Yong Zou, Gary Kobinger, George Fu Gao, Xiangguo Qiu, Songtao Yang, Xianzhu Xia

**Affiliations:** 1 Key Laboratory of Jilin Province for Zoonosis Prevention and Control, Institute of Military Veterinary, Academy of Military Medical Sciences, Changchun, China; 2 Special Pathogens Program, National Microbiology Laboratory, Public Health Agency of Canada, Winnipeg, Manitoba, Canada; 3 CAS Key Laboratory of Pathogenic Microbiology and Immunology, Institute of Microbiology, Chinese Academy of Sciences, Beijing, China; 4 Changchun Institute of Biological Products Co. Ltd., Changchun, China; 5 Center for Disease Control and Prevention, Chengdu Military Region, Kunming, China; 6 Department of Medical Microbiology, Winnipeg, Manitoba, Canada; 7 Department of Immunology, University of Manitoba, Winnipeg, Manitoba, Canada; 8 Department of Pathology and Laboratory Medicine, University of Pennsylvania School of Medicine, Philadelphia, Pennsylvania, USA

## Abstract

Recent successes with monoclonal antibody cocktails ZMapp^TM^ and MIL77 against Ebola virus (EBOV) infections have reignited interest in antibody-based therapeutics. Since the production process for monoclonal antibodies can be prolonged and costly, alternative treatments should be investigated. We produced purified equine antisera from horses hyperimmunized with EBOV virus-like particles, and tested the post-exposure efficacy of the antisera in a mouse model of infection. BALB/c mice were given up to 2 mg of purified equine antisera per animal, at 30 minutes, 1 or 2 days post-infection (dpi), in which all animals survived. To decrease the possibility of serum sickness, the equine antisera was digested with pepsin to generate F(ab′)_2_ fragments, with *in vitro* neutralizing activity comparable to whole immunoglobulin. Full protection was achieved with when treatment was initiated at 1 dpi, but the suboptimal protection observed with the 30 minute and 2 dpi groups demonstrate that in addition to virus neutralization, other Fc-dependent antibody mechanisms may also contribute to survival. Guinea pigs given 20 mg of antisera or F(ab′)_2_ at or starting at 1 or 2 dpi were also fully protected from EBOV infection. These results justify future efficacy studies for purified equine products in NHPs.

Ebola virus (EBOV) is a pathogen from the *Filoviridae* family, and is capable of causing severe hemorrhagic fever in humans and non-human primates. Past outbreaks of EBOV disease (EVD) were sporadic, unpredictable and localized to remote regions of central Africa, with the death toll reaching up to 90%^1^. EBOV is one of the most lethal viruses known to humans and a licensed prophylactic or therapeutic still remains unavailable. In a clinical setting, there is currently little that can be done for infected patients outside of supportive care, which includes fluid replenishment, administration of antivirals, and management of secondary symptoms^2^,^3^. The combination of these reasons means that there are high personal risks involved with working on EBOV in a laboratory setting, and as such it is classified as a Biosafety Level 4 (BSL-4) agent.

In the spring of 2014, a new EBOV variant emerged in the West African nation of Guinea^4^, an area in which the virus had not been previously reported. The outbreak soon spread to neighbouring Sierra Leone and Liberia. Occasionally, cases have been exported into other countries through travel; with countries located in Africa, Europe and North America all having recorded EBOV cases imported by travel, or repatriation of infected citizens. As of mid-December 2015, there are over 11,000 fatalities and 28,000 infections^5^, the largest EVD outbreak in history. Although the outbreak is now largely under control with no reported cases since the week of November 29^th^, 2015^5^, the virus had shown itself at the peak of the outbreak to be extremely resistant to traditional containment methods designed to curb EBOV transmission.

Passive immunotherapy with sera of animal origin has been used for over 120 years to treat bacterial and viral infections, envenomations and drug intoxications. In 2012, a report demonstrated that the passive transfer of IgG from nonhuman primate (NHP) survivors of EBOV disease to naive NHPs was sufficient to confer post-exposure protection against EBOV challenge in all animals^6^. Building on these findings, cocktails of monoclonal antibodies (mAbs) raised against the EBOV glycoprotein (GP) were soon shown afterwards to be effective in the treatment of EBOV disease^7^,^8^. This culminated in the development of ZMapp^TM^, a combination of three mAbs produced in genetically modified tobacco plants, which were shown to reverse advanced EBOV disease in experimentally-infected NHPs^9^, and may have provided a survival benefit when administered to EBOV-infected patients^10^. A second antibody cocktail (MIL-77), which was based on ZMapp^TM^ and produced in modified CHO cells, were later shown to have similar efficacy to ZMapp^TM^ in NHPs^11^. Therefore, passive immunotherapy is an extremely promising approach to control EBOV disease.

Due to the ease of management, high antibody yield and low risk of human contamination by virus or adventitious agents, horses are the most commonly used animal species in the production of hyperimmune sera. Immunization itself is standardized and performed under optimal conditions for both personnel and animals. Passive immunotherapy is still commonly used in countries that are still resource-poor medically, and treatment with immune globulin against rabies virus^12^ and *Clostridium tetani*^13^ infections remains frequent in Africa, Asia and Latin America.

The lower manufacturing costs of hyperimmune equine antisera therefore represents an attractive alternate avenue of treatment, especially to developing and third-world countries, compared to the more costly production process of EBOV GP-specific mAbs. To investigate further, we first prepared purified anti-EBOV antisera via the immunization of horses with EBOV enveloped virus-like particles (eVLP), which consists of EBOV viral protein 40 (VP40) and GP. The equine antisera was then tested *in vitro* using a neutralization assay with recombinant EBOV expressing eGFP (EBOV-eGFP), as well as *in vivo* against a lethal challenge with mouse-adapted EBOV (MA-EBOV) in immunocompetent BALB/c mice. To investigate whether EBOV infections can be controlled by virus neutralization alone, and to prevent the possible induction of serum sickness in humans that would be administered antisera, the post-exposure efficacy of F(ab′)_2_ (immunoglobulin treated with pepsin to remove the Fc regions of the antibody) were also investigated side-by-side with equine antisera in all experiments as a potential alternate treatment. Guinea pigs then were used to confirm the efficacy results from mouse studies, due to their status as a higher phylogenic species which more closely models hallmarks of EVD in humans. Additionally, prior to efficacy studies both the equine antisera and F(ab′)_2_ had been evaluated for safety in the Peking Union Medical College Center for New Drug Safety Evaluation, Chinese Academy of Medical Sciences, which is certified by the Food and Drug Agency of the People’s Republic of China. Both equine-derived products were found to meet safety standards for clinical use in China.

## Results

### Immunization of horses and production of equine antibody products

**Figure 1:**
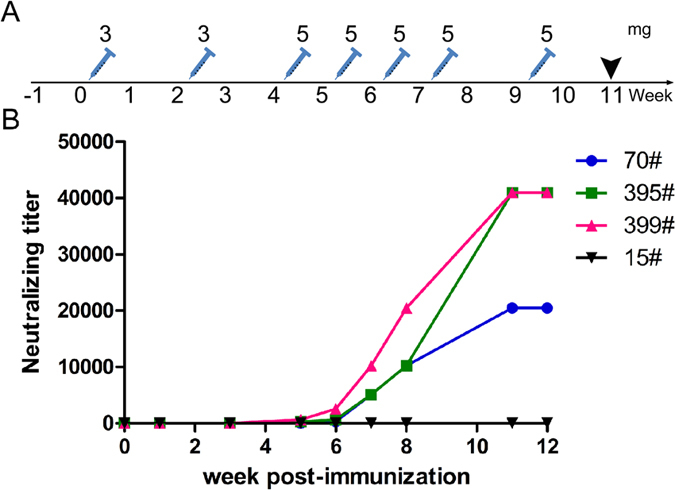
Horse immunization procedure and neutralizing antibody titers. **(A)** Horses (70#, 395#, 399#) were vaccinated IM with 3 mg of eVLP (at 0 and 2 weeks) or 5 mg of eVLP (at 4, 5, 6, 7, 9 weeks), and horse 15# was vaccinated with equal quantity of insect cell protein, and hyperimmune sera were collected at 11 weeks after the first vaccination. (**B)** An inhibition assay with EBOV GP-pseudotyped HIV-1 virus was conducted to determine the neutralizing antibody titers of 0, 1, 3, 5, 6, 7, 8, 11 weeks. Numbers 70, 395 and 399 represent individual horses that were vaccinated, and number 15 represents a control horse. Assays were repeated three times in triplicate, and the results presented (from one assay) are representative of all three assays.

**Figure 2:**
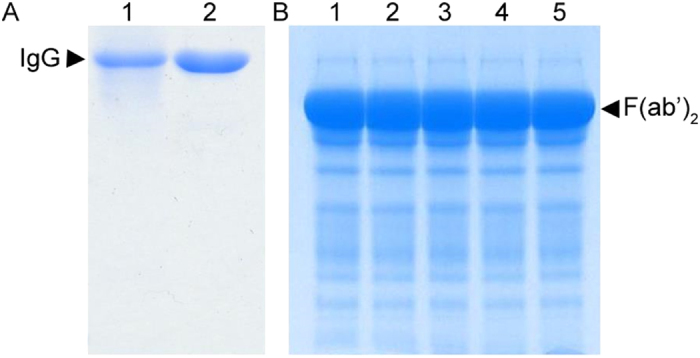
Purity analysis of antisera and F(ab′)_2_. Coomassie blue staining of antisera and F(ab′)_2_ preparations. 10 μg of antisera and F(ab′)_2_ were mixed with non-reducing (without β-mercaptoethanol) protein sample buffer, heated at 95 °C for 5 min, and then subjected to SDS-PAGE followed by Coomassie blue staining. (**A)** antisera preparations (lane 1–2). The purity was determined to be 95%. (**B)** F(ab′)_2_ preparations (lane 1–5). The purity was determined to be 83%.

The horses were immunized with eVLP produced from the infection of Sf9 cells with rBV-VP40-GP. The filamentous eVLP were observed under an electron microscope (Supplementary Figure 1) and confirmed to be morphologically similar to EBOV. Immunoblotting of rBV-VP40-GP infected Sf9 cell lysates demonstrated that the eVLP contained EBOV GP and VP40 (Supplementary Figure 2). Three horses were immunized intramuscularly (IM) with 7 injections of eVLP over 11 weeks and the hyperimmune sera were collected from each animal at specified timepoints (Fig. 1A) to determine the serum titers by neutralization assay against a recombinant HIV-1 virus pseudotyped with EBOV GP. A pseudotyped-virus was used for these studies to confirm the *in vitro* efficacy of the antisera preparations under Biosafety Level 2 (BSL-2) conditions, before subsequent studies in the BSL-4 laboratory. Neutralizing serum titers were detectable after the 5th immunization at week 6, and increased until the 7th immunization at week 11 (Fig. 1B). The serum neutralizing antibody titers of two horses were 1:40,960 after seven immunizations, and was 1:20,480 for the third animal. The equine antisera (purified by ammonium sulphate-based precipitation) was then digested with pepsin at a concentration of 5–10 U/ml for 45 to 90 min and purified to generate F(ab′)_2_ fragments. The purity of the antisera and F(ab′)_2_ preparations, as determined by SDS-PAGE followed by thin layer chromatography, was determined to be 95% and 83%, respectively (Fig. 2). Approximately 2000 mL of purified antisera could be obtained from each horse during each collection, with the stock concentration between 20–25 mg/mL. Up to 3–5 collections can be performed on each horse, therefore each hyperimmunized horse yields between 120 to 250 g of purified antisera.

### *In vitro* characterization of equine antisera and F(ab‘)_2_

**Figure 3:**
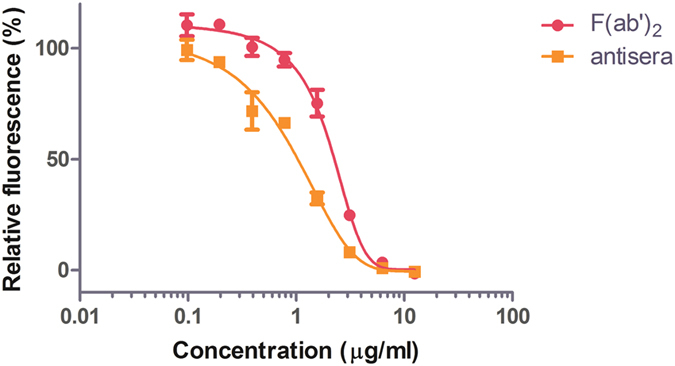
Neutralizing antibody titers of F(ab′)_2_ and antisera. The neutralizing activities of equine antisera and F(ab′)_2_ against EBOV-eGFP were compared over different concentrations (x-axis). Total fluorescence from infected VeroE6 cells at 3 dpi were shown as a percentage of the fluorescence observed with the PBS control, which is set at 100% (y-axis). Samples were processed in triplicate, and error bars indicate standard error. Data shown in this figure are representative of three independent neutralization studies with F(ab′)_2_ or antisera.

The equine antisera and F(ab′)_2_ products were first characterized against a laboratory generated EBOV-eGFP for their potential to neutralize EBOV. Equine antisera and F(ab′)_2_ were found to possess levels of similar neutralizing activity, with the half effective maximal concentration (EC_50_) of the products to be 1.07 μg/mL and 2.12 μg/mL, respectively (Fig. 3). Moreover, the neutralization activities of both the equine antisera and F(ab′)_2_ were complete at a concentration of 12.5 μg/mL, indicating that the two products were indistinguishable from each other in terms of neutralization at high concentrations. EBOV-specific IgG in the equine antisera preparations were also determined by ELISA. Serial 2-fold dilutions of stock antisera with a concentration of 18–20 mg/mL were assayed in triplicate over three independent experiments, and the titer was determined to be between 10,240 and 20,480 endpoint dilutions.

### *In vivo* characterization of equine antisera and F(ab‘)_2_

**Figure 4:**
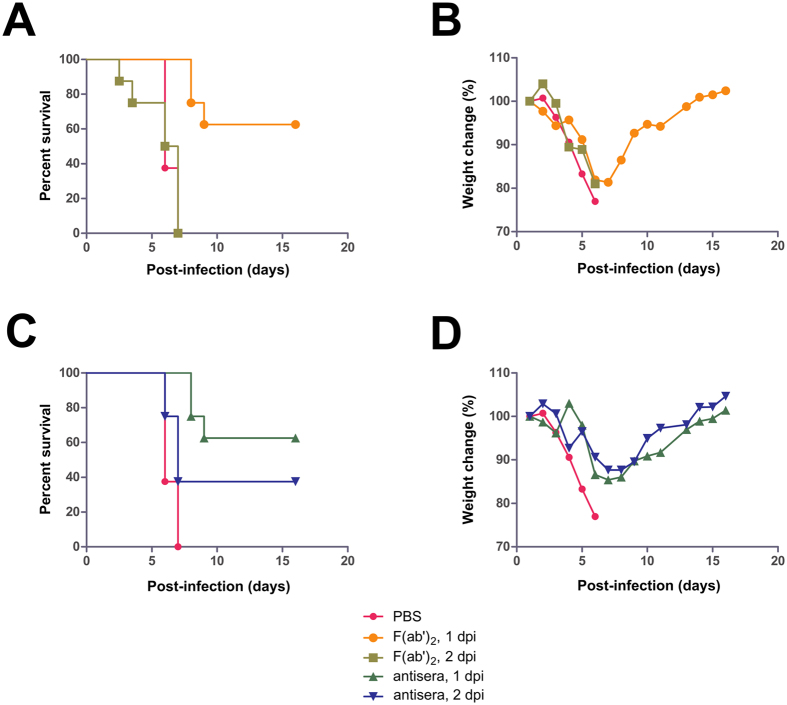
Experimental findings of mice given equine antisera or F(ab′)_2_. Groups of 8 BALB/c mice were administered IP with either F(ab′)_2_, twice daily for 3 days starting at 1 or 2 dpi with MA-EBOV, or one injection of antisera at 1 or 2 dpi. Injections contained 200 μg of antisera or F(ab′)_2_. (**A)** The survival and (**B)** percentage weight change are shown for mice administered F(ab′)_2_, whereas (**C)** the survival and (**D)** percentage weight change are shown for animals given equine antisera.

The half-life of the F(ab′)_2_ and equine antisera were first assessed in guinea pigs, and found to be 8–12 hours and 3–4 days, respectively (Supplementary Table 1). The protective efficacy of equine antisera and F(ab′)_2_ were assessed over three experiments in BALB/c mice. The first experiment was to compare the efficacy of antisera and F(ab′)_2_ treatments side-by-side. Groups of 8 mice were given intraperitoneal (IP) injections of F(ab′)_2_ at 200 μg per dose, twice daily for 3 days (b.i.d. × 3d), starting at either 1 or 2 days post-infection (dpi) with a uniformly lethal dose of MA-EBOV. For comparison, groups of 8 mice were administered an IP injection of equine IgG at 200 μg per dose (q.d. × 1d), at either 1 or 2 dpi. Control mice were given an equal volume of PBS in place of the treatment (q.d. × 1d) at 1 dpi. PBS was used instead of non-specific immunoglobulin or F(ab′)_2_, because previous studies involving passive transfer of non-specific antisera in mice and NHPs did not result in protection^14^,^15^, and thus survival due to the non-specific effects of serum proteins is considered unlikely. Control mice lost approximately 23% of its body weight over the course of the experiment and none survived, with a mean time to death (MTD) of 6.4 ± 0.5 dpi. Five of eight mice given F(ab′)_2_ starting at 1 dpi survived (p-value < 0.0001, compared with PBS group), with an average weight loss of 18.7% and a MTD of 8.3 ± 0.6 dpi; however, none of the mice given F(ab′)_2_ starting at 2 dpi survived the challenge (p-value = 0.9777, compared with PBS group), with an average weight loss of 19.0% and a MTD of 5.8 ± 1.8 dpi (Fig. 4A,B). Five of eight mice given antisera at 1 dpi survived (p-value < 0.0001, compared with PBS group), with an average weight loss of 14.6% and a MTD of 8.3 ± 0.6 dpi (Fig. 4C). Three of eight mice given antisera at 2 dpi survived (p-value = 0.0446, compared with PBS group), with an average weight loss of 12.3% and a MTD of 6.6 ± 0.6 dpi (Fig. 4D). Comparing groups with equal treatment times, there was no statistical difference between F(ab′)_2_ at 1 or 2 dpi (p-value 1.0000 and 0.0613, respectively). However, given that multiple administrations of F(ab′)_2_ were required to achieve similar protection levels demonstrated by a single injection of antisera, the results suggest that equine antisera is a superior product to F(ab′)_2_ in terms of efficacy, possibly due to a longer *in vivo* half-life of equine antiseras.

**Figure 5:**
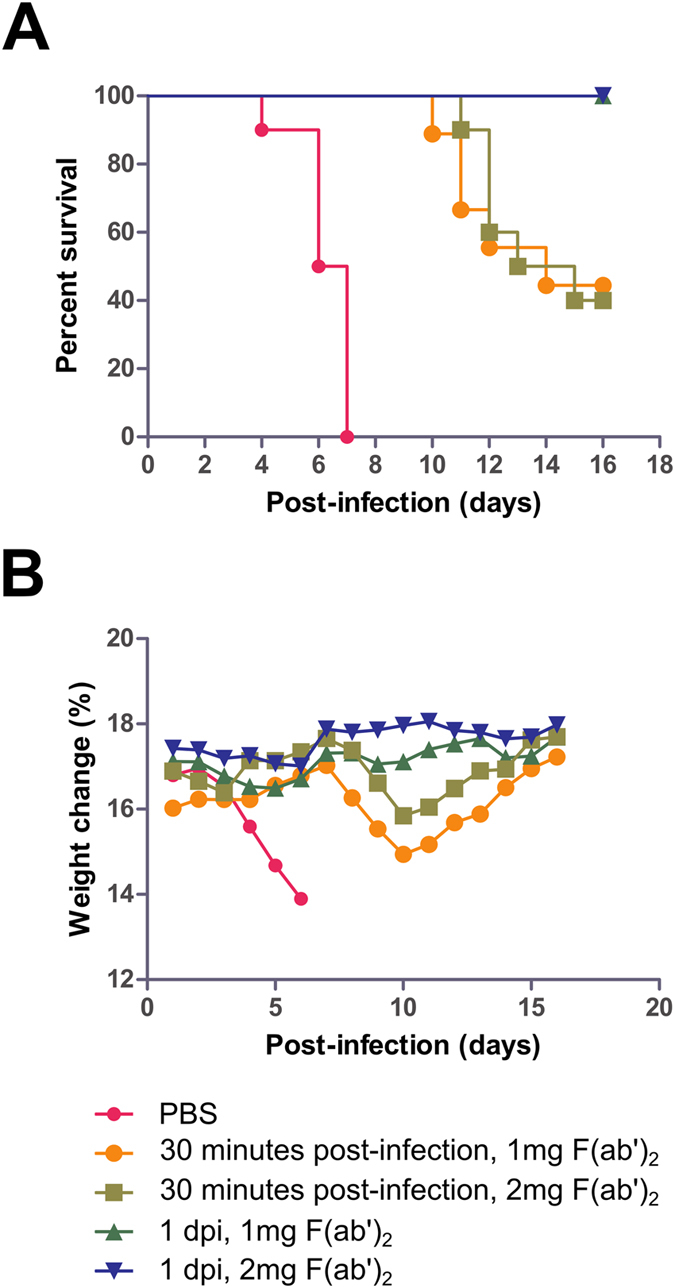
Experimental findings of mice given equine F(ab′)_2_. Groups of 9–10 BALB/c mice were administered IP with F(ab′)_2_, twice daily for 3 days starting at 30 minutes or 1 dpi with MA-EBOV, at a dose of either 1 or 2 mg F(ab′)_2_. (**A)** The survival and (**B)** percentage weight change are shown for both treatments.

Since F(ab′)_2_ appear to be promising early in MA-EBOV infection, the dosage of this treatment was increased for a second experiment. Groups of 9–10 mice were given IP injections of F(ab′)_2_ at 1 or 2 mg per dose, twice daily for 3 days (b.i.d. × 3d), starting at either 30 minutes or 1 dpi with MA-EBOV. Control mice were treated with PBS (q.d. × 1d) at 1 dpi. Control mice lost approximately 17.3% of its body weight over the course of the experiment and none survived, with a MTD of 6.3 ± 1.0 dpi. Partial survival was observed when treatment began 30 minutes after challenge. Four of nine mice in the 1 mg group survived (p-value < 0.0001, compared with PBS group), with an average weight loss of 6.8% and a MTD of 11.6 ± 1.5 dpi, whereas four of ten mice in the 2 mg group survived (p-value < 0.0001, compared with PBS group), with an average weight loss of 6.2% and an MTD of 12.5 ± 1.4 dpi (Fig. 5A,B). In contrast, all mice survived if treatment was initiated at 1 dpi, with negligible weight loss (less than 5%) observed in both the 1 and 2 mg groups (p-value < 0.0001, compared with PBS group), indicating that protection was complete. These results indicate that F(ab′)_2_ can contribute to protection from MA-EBOV, but only within a certain timeframe after challenge.

**Figure 6:**
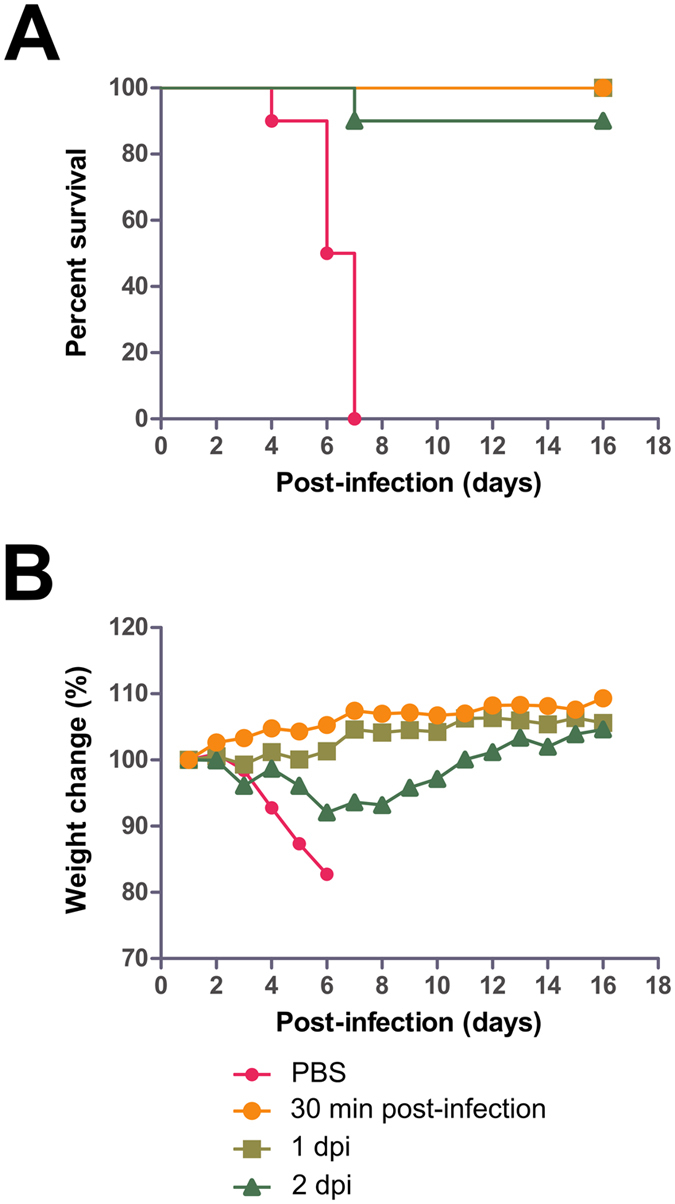
Experimental findings of mice given equine antisera. Groups of 10 BALB/c mice were administered IP with 2 mg of antisera, at 30 minutes, 1 or 2 dpi with MA-EBOV. (**A)** The survival and (**B)** percentage weight change are shown for both treatments.

The efficacy of equine antisera at higher doses was then investigated in a third experiment. Groups of 10 mice were given IP injections of antisera at 2 mg per dose (q.d. × 1d), at 30 minutes, 1 or 2 dpi with MA-EBOV. Control mice were treated with PBS (q.d. × 1d) at 1 dpi. Control mice lost approximately 17.3% of its body weight over the course of the experiment and none survived, with a MTD of 6.3 ± 1.0 dpi. All mice treated with antisera at 30 minutes and at 1 dpi survived with negligible weight loss (p-value < 0.0001, compared with PBS group). Nine of ten mice treated with antisera at 2 dpi also survived the challenge (p-value < 0.0001, compared with PBS group), with the lone non-survivor dying at 7 dpi and the average peak weight loss determined to be 7.9% within this treatment group (Fig. 6A,B). These results again indicate that equine antisera, compared to F(ab′)_2_ is able to offer a greater contribution to protection from MA-EBOV in the mouse model due to the need for fewer administrations to achieve a similar level of protection.

**Figure 7:**
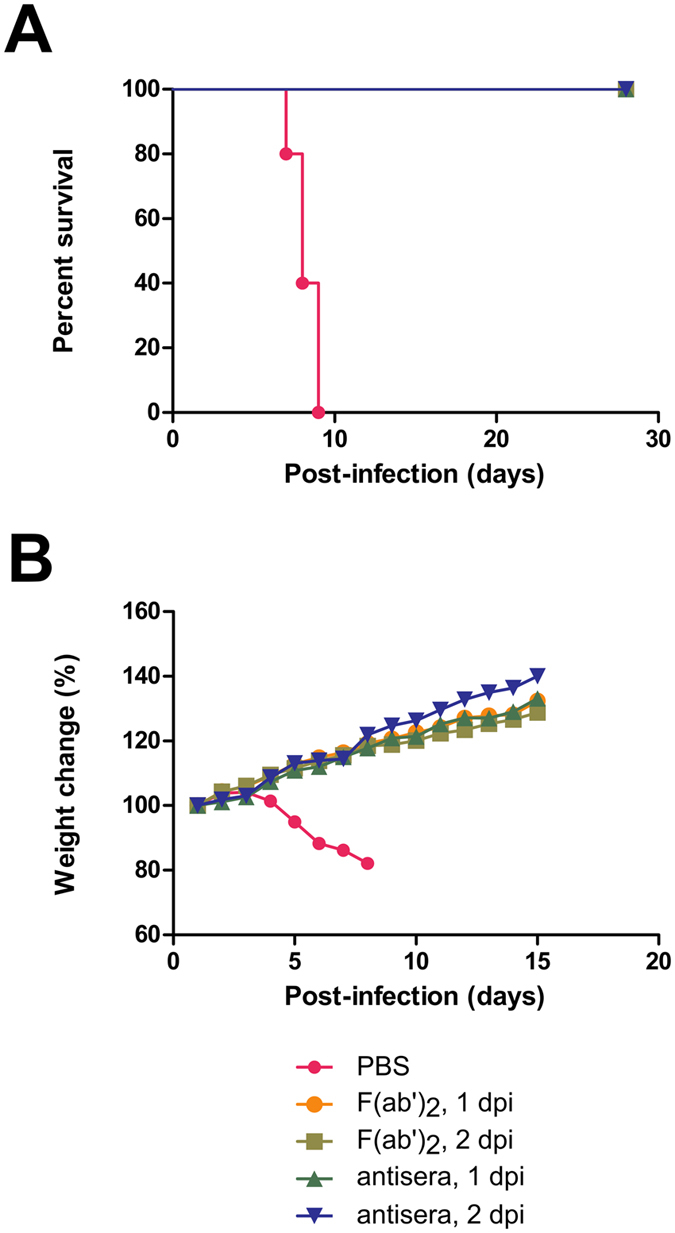
Experimental findings of guinea pigs given equine antisera or F(ab′)_2_. Groups of 6 guinea pigs were administered IP with 20 mg of antisera or F(ab′)_2_, at 1 or 2 dpi with 1000 × LD_50_ GA-EBOV. (**A)** The survival and (**B)** percentage weight change are shown for both treatments. A group of 5 control animals were given PBS as a mock treatment.

Guinea pigs were then used to confirm the protective effects from antisera and F(ab′)_2_ in a higher phylogenic species for EBOV challenge, since these animals better mimic hallmarks of human EBOV infections, such as coagulation abnormalities^16^. Groups of 6 animals were given a single IP injection of antisera (q.d. × 1d) starting at 1 or 2 dpi with GA-EBOV, or twice-daily IP injections of F(ab′)_2_ for three days (b.i.d. × 3d), starting at 1 or 2 dpi. Each injection dose contained 20 mg antisera or F(ab′)_2_. A group of five control animals were given a single injection of PBS at 1 dpi. Control guinea pigs lost approximately 17.9% of its body weight over the course of the experiment and none survived, with a MTD of 8.2 ± 0.6 dpi. All guinea pigs that were given antisera at 1 or 2 dpi survived (p-values = 0.0011 for both groups, compared with PBS group), with no clinical signs of disease or significant weight loss (Fig. 7A,B). In addition, animals that were given F(ab′)_2_ starting at 1 or 2 dpi survived GA-EBOV challenge (p-values = 0.0011 for both groups, compared with PBS group) without signs of disease or significant weight loss (Fig. 7A,B). The disparity between the efficacy of F(ab′)_2_ between mice and guinea pigs may be attributed to a much higher dosage of F(ab′)_2_ administered per weight: each F(ab′)_2_ dose for guinea pigs was 100× higher than those given to mice, but the guinea pigs used in this experiment were only 10–20 times bigger than the mice by weight.

## Discussion

The lack of BSL-4 laboratories globally, trained personnel as well as the rigors of working under high biocontainment conditions have severely hampered basic research with EBOV, leading to a dearth of vaccines and therapeutics for use in humans. These weaknesses were exposed with the 2014–15 EBOV crises, and highlight the value and need for basic and translational science that occurs prior to an impending threat. In an effort to save lives, several experimental candidate treatments that had been tested for efficacy in mice or NHPs, in addition to approved or nearly-approved drugs developed against unrelated pathogens, were expedited for use in humans under compassionate circumstances, with varying degrees of success^17^. Of these, antibody-based treatments appear to hold the most promise in the clinic and thus should be further investigated for use in humans.

Passive immunotherapy against EBOV had been tried in the past. Towards the end of the 1995 EBOV outbreak in Kikwit, Zaïre (presently Democratic Republic of the Congo), the passive transfer of whole blood from EBOV survivors to patients resulted in the survival of seven out of eight recipients^18^. However, safety concerns with blood transfusions, such as the spreading of blood-borne diseases, allergic reactions and concerns of inefficacy^19^ mean that this approach is controversial and will likely not be used unless a better alternative was unavailable. In the 1990s, Russian investigators had prepared hyperimmunized antisera from horses vaccinated with inactivated EBOV, which were shown to be effective when administered at a dose of 1 mg/kg to baboons, with 100% survival (2 survivors out of 2) when given 2 hours before challenge, and 67% (2 survivors out of 3) when given immediately after challenge^20^, although baboons are known to be more resistant to EBOV infection compared to other NHP species^21^. The survival benefits of the equine antisera were limited to a delay in the onset of viremia clinical symptoms when tested in cynomolgus macaques immediately after challenge, at a dose of 60 mg for an animal weighing between 5–6 kg^22^. Comparing to successful passive immunotherapy studies with purified IgG from NHPs (80 mg/kg), as well as ZMapp and MIL-77 (50 mg/kg), the dosage for the cynomolgus macaque experiment was likely too low at the time. Use of equine antisera as an emergency post-exposure treatment against EBOV has been approved in Russia, and several investigators that had been accidentally exposed to the virus have been administered this treatment, although it is not clear if they had actually been infected^23^.

The results of this study show that both equine antisera and F(ab′)_2_ preparations, which can be rapidly produced in large quantities at a lower cost compared to mAbs, are effective in the post-exposure treatment of MA-EBOV challenged mice and GA-EBOV infected guinea pigs. F(ab′)_2_ was developed in this study due to past documented issues with antisera administrations, in which equine botulinum toxin and anti-snake venom antisera was shown to produce serum sickness at high doses^24^. However, mice that were given a single dose of antisera demonstrated more complete protection over a greater period of time compared to multiple injections of F(ab′)_2_. This suggests that F(ab′)_2_ has a shorter half-life compared to IgG (which has been confirmed in this study), and/or that virus neutralization plays a partial role in survival from EBOV. The observation that administering F(ab′)_2_ at 1 dpi is more efficacious than when the same treatment was given at 30 minutes post-exposure (Fig. 5A) was also observed in past studies with therapeutic EBOV GP-specific monoclonal antibodies^25^,^26^, and suggests that virus neutralization may play a bigger role in protection at a later timepoint after EBOV challenge. With regards to neutralization, past reports have shown that while elevated antisera levels in NHPs vaccinated against EBOV correlated with survival, levels of specific neutralizing antibodies did not^27^. It is a possible explanation as to why mAb KZ52, which was originally selected for its ability to neutralize EBOV^28^, failed to protect NHPs when given at a dose of 50 mg/kg starting 1 day before challenge^29^, and suggests that in the future, the screening of potentially efficacious antibodies against EBOV should not be based solely on the ability of the antibody to neutralize virus.

Fc-dependent antibody functions, which include antibody dependent cellular cytotoxicity, complement-dependent cytotoxicity, and neonatal Fragment crystallizable receptor (FcR)-mediated cross-presentation, likely play a role in protection. For instance, studies in mice against Yellow Fever virus and West Nile virus infections have shown that the protective mechanisms of monoclonal antibodies are dependent on FcR^30^,^31^. Furthermore, complement component C1q has been previously shown in Influenza virus and West Nile virus infections to directly enhance the neutralizing activities of antibodies^32^,^33^. These mechanisms are not mutually exclusive, but determining their relative importance of each proposed mechanism to efficacy and survival will yield further insight into the specific mechanisms in which antibodies help protect against EBOV disease. Furthermore, the findings of this study justify the testing of equine IgG in a higher phylogenic species for EBOV, such as NHPs, and may result in the development of a safe and economical method for the production of an effective EBOV therapeutic.

## Materials and Methods

### Ethics statement

Mice and guinea pig experiments were performed at the BSL-4 laboratory in the National Microbiology Laboratory (NML) in Winnipeg, Canada. All rodent experiments have been approved by the Animal Care Committee (ACC) at the Canadian Science Center for Human and Animal Health (CSCHAH), in accordance with the guidelines outlined by the Canadian Council on Animal Care (CCAC). Horse studies were conducted with prior approval from the Animal Welfare and Ethics committee of the Institute of Military Veterinary, Academy of Military Medical Sciences (permit number SCXK-2012-017), according to Horse Quarantine and Immunization Protocols for Equine Serum Production.

### Animals

BALB/c mice and Hartley guinea pigs were purchased from a commercial supplier (Charles River). The animals were kept in sterile, autoclaved cages and provided sterilized food and water *ad libitum*. Animals were also provided red, plastic shelters inside the cages as an added source of stimulation. Horses were purchased from a commercial supplier (Red Hill Military horse farm) and provided food and water *ad libitum*.

### Construction and generation of recombinant baculoviruses

The sequences of EBOV GP and VP40, with lengths of 2,031 and 981 bp, respectively, were derived from GenBank (Zaire ebolavirus strain Zaire 1995, complete genome; GenBank accession No. AY354458). The GP and VP40 genes were cloned into the pUC-57 vector, named pUC-GP and pUC-VP40, and then inserted into pFastBac Dual vector (Life technologies) under the polyhedrin promoter and p10 promoter respectively, resulting in plasmid pFastBac Dual-VP40-GP. The purified plasmid was transformed into DH10™Bac *E. coli* (Life technologies) for transposition into a bacmid. A Cellfection^®^ II Reagent (Life technologies) was used according to manufacturer instructions to generate recombinant baculoviruses co-expressing Ebola VP40 and GP (rBV-VP40-GP).

### eVLP production and inspection

To produce eVLP, Sf9 cells were infected with rBV-VP40-GP at a multiplicity of infection (MOI) of 1, and incubated at 27 °C for 4 days. Culture supernatants were harvested and centrifuged at 2000 × g for 30 min to remove cells, and then pelleted by ultracentrifugation at 30,000 × g for 60 min at 4 °C. The pellets were re-suspended in PBS and purified through a 10–30–50% discontinuous sucrose gradient at 25,000 × g for 90 min at 4 °C. The eVLP band obtained between 30% and 50% density range was collected, washed, and re-suspended in PBS^34^.

For immunoblotting, eVLP and control sample (cell culture supernatant) were separated by 12% sodium dodecyl sulfate-polyacrylamide gel electrophoresis under denaturing conditions, transferred onto a polyvinylidene fluoride (PVDF) membrane (Whatman, Kent, UK) and then probed with mouse anti-EBOV GP polyclonal antibody (Prokaryotic expression GP protein immunized mice) or mouse anti-EBOV VP40 monoclonal antibody (ab1918, abcam) at a dilution of 1:200 overnight at 4 °C. The sample was then incubated with horseradish peroxidase (HRP)-conjugated goat anti-mouse secondary antibody at a dilution of 1:4000 (Millipore, Boston, MA, USA) for 60 min at 37 °C. The PVDF membrane was colored with a chemiluminescence solution (Pierce Biotechnology, Rockford, IL, USA).

For electron microscopy, eVLP were applied onto a carbon-coated formvar grid, which was immediately stained with 1% phosphotungstic acid and then observed by a transmission electron microscope.

### Horse immunizations

Horses (n = 3) were vaccinated intramuscularly (subcutaneous multi-point injection) with either 3 or 5 mg of eVLPs emulsified in Freund’s complete adjuvant/Freund’s incomplete adjuvant at weeks 0, 2, 4, 5, 6, 7 and 9 (7 times). Blood samples were collected from the jugular vein at 1 week prior to the first immunization and 1 week after each immunization, and the sera were stored at −80 °C until further analysis.

### Fragmentation and purification of equine antibody products

The equine antisera and F(ab′)_2_ were produced at Changchun Institute of Biological Products Co., Ltd., using the large-scale, Current Good Manufacturing Practice compatible equine antiserum manufacturing platform. Equine antisera and F(ab′)_2_ preparations were characterized according to guidelines set forth by *Chinese Pharmacopoeia* (2010 edition), including appearance, color, visible foreign matter, pH value, F(ab′)_2_ and immunoglobulin content, and sterility. For equine antisera, the blood was taken from the jugular vein of immunized horses, and the sera were separated 2 h later. The horse sera were diluted 8-fold with PBS, centrifuged at 12,000 rpm for 30 min, and supernatants were subjected to filtration through a 0.45 μm filter. The antisera were purified by ammonium sulphate precipitation^35^, followed by salt column, and then stored in 0.9% NaCl solution. For F(ab′)_2_: horse sera were diluted 3-fold and the pH was adjusted to 3.2 using 1 M HCl, and then the antisera were subjected to shock digestion at 37 °C for 30 min with pepsin, and 0.4 M NaOH was added to terminate digestion. The digestion products were subjected to salt column purification, followed by Protein A column. The flow-through fluid was harvested and subjected to ultrafiltration to remove the pepsin and small molecular proteins, and the F(ab′)_2_ was stored in 0.9% NaCl solution.

### SDS-PAGE and thin layer chromatography

The purified equine antisera and F(ab′)_2_ samples were mixed with non-reducing (without β-mercaptoethanol) protein sample buffer, heated at 95 °C for 5 min, and then subjected to SDS-PAGE (12% gel, staining for 3 h and destaining for 2 h); and then the target fractions in the gel were examined by thin layer chromatography scanner (CS-9301, Shimadzu), (transmission, zigzag scan, dual wavelength, swing width: 8 mm, delta Y: 0.1 mm) to determine the purity of equine antisera and F(ab′)_2_.

### ELISA

EBOV GP expressed by *E. coli* BL-21 was diluted with 0.1 M bicarbonate buffer pH 9.6 to a final concentration of 10 μg/mL, and coated on 96-well ELISA plates overnight at 4 ^o^C (100 μL/well). After blocking with Angiotensin Converting Enzyme (ACE) buffer (Bio-Rad, California, USA) at 37 °C for 2 h, the plates were incubated with 100 μL of serial 2-fold dilutions of stock equine antisera in triplicate at 37 °C for 1.5 h. After washing, 100 μL of HRP-conjugated goat anti-horse IgG (ComWin Biotech Co., Ltd. Beijing, China) diluted 1:20000 in PBS-0.5% Tween was added to the wells and incubated at 37 °C for 50 min. After washing, 100 μL of substrate 3′,5,5′-Tetramethylbenzidine (TMB, Sigma) was added and incubated at room temperature for 30 min. The reaction was stopped by adding 50 μL of 0.5 M H_2_SO_4_, and the absorbance was measured at 450 nm. A dilution was considered positive if the absorbance reading was at least twice that of the negative control (PBS) at the same dilution. The IgG endpoint titer was calculated as the highest dilution still showing a positive result. The assay was performed independently three times.

### Half-life studies in guinea pigs

Group of 4 guinea pigs were administered an IP injection of 1 mL purified equine antisera (neutralization titer 1:20480) or F(ab′)_2_ (neutralization titer 1:20480). Blood samples were collected at 0, 1, 4, 8, 12, 24, 48, 72, 96, 120, 144 h post-injection, and sera were used for determination of neutralization titer against a recombinant HIV-1 virus pseudotyped with EBOV GP. The time range post-infection in which the titer decreases by 50% is considered the half-life. Each sample was performed in triplicate.

### Determining the protective efficacy of equine antisera

BALB/c mice, 4–6 week old, female and weighing between 15–19 g, were randomly assigned into groups of 8–10 mice. All animals were challenged intraperitoneally (IP) with a dose of 1000 × LD_50_ mouse-adapted EBOV (MA-EBOV, USAMRIID/BALB/c-lab/COD/1976/Mayinga-MA-p3) in 200 μL DMEM. The treatment was given IP (q.d. × 1d) at 1 or 2 days post-infection (dpi) with 0.2 mg equine anti-EBOV antisera per mouse, or in a subsequent experiment, given IP at 30 minutes, 1 or 2 dpi with 2 mg equine anti-EBOV antisera per mouse. The control group was given the same volume of PBS as mock treatment. All animals were monitored for signs of disease, survival and weight change for 16 days, and survival was monitored for 12 additional days. Female strain Hartley guinea pigs, 4–6 week old and weighing between 200 and 300 g, were randomly assigned into groups of 6 animals. All animals were challenged IP with 1000 × LD_50_ guinea pig-adapted EBOV (GA-EBOV, VECTOR/C.porcellus-lab/COD/1976/Mayinga-GPA-p7) in 1 mL DMEM. The treatment was given IP (q.d. × 1d) at 1 or 2 dpi with 20 mg equine anti-EBOV IgG per animal. A control group of 5 guinea pigs were given the same volume of PBS as mock treatment. All animals were monitored for signs of disease, survival and weight change for 15 days, and survival was monitored for 13 additional days.

### Determining the protective efficacy of F(ab′)_2_

BALB/c mice, 4–6 week old, female and weighing between 15–19 g, were randomly assigned into groups of 8–10 mice. All mice were challenged IP with a dose of 1000 × LD50 MA-EBOV in 200 μL DMEM. The treatment was given IP at 1 or 2 dpi with 200 μg F(ab′)_2_ per mouse, or in a subsequent experiment, given IP at 30 minutes, 1 or 2 dpi with either 1 mg or 2 mg of F(ab′)_2_ per mouse. The treatment was given via IP twice a day for 3 days (b.i.d. × 3d). The control group was given the same volume of PBS as a mock treatment. All animals were monitored for signs of disease, survival and weight change for 16 days, and survival was monitored for 12 additional days. Female strain Hartley guinea pigs, 4–6 week old, were randomly assigned into groups of 6 animals. All animals were challenged IP with 1000 × LD_50_ guinea pig-adapted EBOV in 1 mL DMEM. The treatment was given IP at 1 or 2 dpi with 20 mg F(ab′)_2_ per animal. The treatment was given via IP twice a day for 3 days (b.i.d. × 3d). A control group of 5 guinea pigs were given the same volume of PBS as mock treatment. All animals were monitored for signs of disease, survival and weight change for 15 days, and survival was monitored for 13 additional days.

### Pseudotyped virus neutralization assay

Titers of equine antisera and F(ab′)_2_ from horses was tested in an neutralization assay in Huh-7 cells against recombinant HIV-1 virus pseudotyped with EBOV GP. The method for generating pseudotyped viruses was described in a previous publication^36^. Briefly, pseudotyped virus containing supernatants were incubated either with serially diluted horse sera at 37°C for 1 h, before addition to pre-plated target cells in 96-well culture plates (density of 10^4^ cells/well). Cells were re-fed fresh medium 4 h after addition, and followed by lysing cells at 48 h using cell lysis buffer (Promega) and transferring the lysates into 96-well luminometer plates. Luciferase substrate (Promega) was added to the plates, and the relative luciferase activity was determined. The inhibition of pseudotyped was presented as % inhibition. The highest serum dilution giving over 50% reduction of luciferase activity was regarded as the neutralizing antibody titer.

### EBOV-eGFP neutralization assay

Serial two-fold dilutions of F(ab′)_2_ or antisera (between 12.5 to 0.097656 μg/mL) were incubated with 100 plaque forming units (PFU) of EBOV expressing eGFP (EBOV-eGFP, NML/H.sapiens-lab/COD/1976/Mayinga-eGFP-p3) at 37 °C for 1 h, transferred to Vero E6 cells and incubated at 37 °C for 1 h, and then replaced with DMEM supplemented with 2% fetal bovine serum. Control wells contained PBS instead of F(ab′)_2_ or antisera, and all samples were repeated in triplicate. The plates were fixed with 10% phosphate buffered formalin at 72 h after infection, and scored for the intensity of eGFP using the BioTek Synergy HT microplate reader. The results were expressed as a percentage of the fluorescence reading with the control (which is set at 100%), and fitted to a 4-parameter logistic curve (Graphpad).

### Statistical analysis

The p-values for rodent studies were determined using the Log-rank (Mantel-Cox) test. Calculated values of less than 0.05 were considered statistically significant. All *in vivo* studies were performed once.

## Additional Information

**How to cite this article**: Zheng, X. *et al*. Treatment with hyperimmune equine immunoglobulin or immunoglobulin fragments completely protects rodents from Ebola virus infection. *Sci. Rep.* **6**, 24179; doi: 10.1038/srep24179 (2016).

## Supplementary Material

Supplementary Information
