# Supplementary material for: Treatment with hyperimmune equine immunoglobulin or immunoglobulin fragments completely protects rodents from Ebola virus infection
Source: Sci Rep. 2016 Apr 12;6:24179. doi: 10.1038/srep24179 (PMC4828711; doi:10.1038/srep24179)
Supplement: Supplementary Information [file srep24179-s1.pdf]

Supplementary data

Manuscript title:

Treatment with hyperimmune equine immunoglobulin or immunoglobulin fragments completely protects rodents from Ebola virus infection

Author list:

Xuexing Zheng, Gary Wong, Yongkun Zhao, Hualei Wang, Shihua He, Yuhai Bi, Weijin Chen, Hongli Jin, Weiwei Gai, Di Chu, Zengguo Cao, Chong Wang, Quanshui Fan, Hang Chi, Yuwei Gao, Tiecheng Wang, Na Feng, Feihu Yan, Geng Huang, Ying Zheng, Nan Li, Yuetao Li, Jun Qian, Yong Zou, Gary Kobinger, George Fu Gao, Xiangguo Qiu, Songtao Yang, Xianzhu Xia

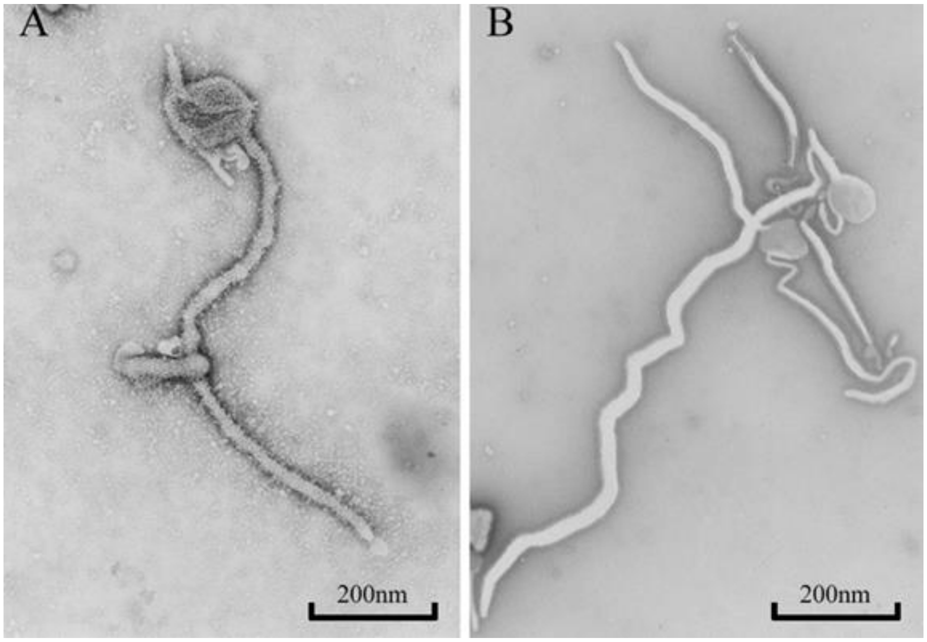

Supplementary Figure 1. Electron microscopy of eVLP. Shown are electron micrographs of eVLP. (A) or (B) at × 25,000. Particles, obtained by ultracentrifugation of the supernatants of Sf9 cells infected with rBV-VP40-GP, were negatively stained with uranyl acetate to reveal the ultrastructure.

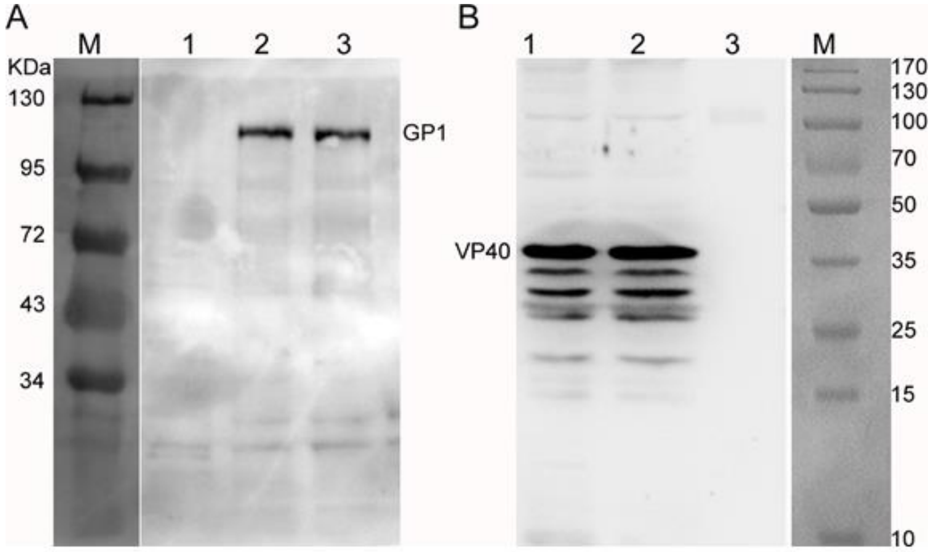

Supplementary Figure 2. Western blot analysis of eVLP preparations. Sf9 cells were infected with rBV-GFP (control protein) or rBV-VP40-GP. The eVLP preparations were resuspended in PBS at the concentration of 5 μg of eVLP preparations or control protein were mixed with reducing protein sample buffer, heated at 95 °C for 5 min, and then subjected to SDS-PAGE followed by Western blot analysis using a polyclonal antibody anti-GP (A) or monoclonal antibodies anti-VP40 (B). A: Control protein (lane 1); eVLP (lane 2-3). B: eVLP (lane 1-2); Control protein (lane 3). Both the GP and VP40 protein bands were present in eVLP sample. Equal quantity of proteins was loaded into each lane.

Supplementary Table 1. Determination of half-life of purified equine antisera and F(ab')$_2$ in guinea pigs

Group	Guinea pig number	Neutralization titer										
		0h	1h	4h	8h	12h	24h	48h	72h	96h	120h	144h
purified antisera	1	-	1:160	1:160	1:320	1:320	1:320	1:320	1:320	1:160	1:160	1:80
	2	-	1:80	1:80	1:160	1:160	1:160	1:160	1:160	1:80	1:80	1:40
	3	-	1:160	1:160	1:320	1:320	1:320	1:320	1:160	1:160	1:80	1:80
	4	-	1:160	1:160	1:160	1:160	1:160	1:160	1:80	1:80	1:80	1:40
F(ab')$_2$	1	-	1:160	1:320	1:320	1:160	1:80	-	-	-	-	-
	2	-	1:160	1:160	1:160	1:80	1:40	-	-	-	-	-
	3	-	1:160	1:160	1:160	1:80	-	-	-	-	-	-
	4	-	1:160	1:160	1:80	1:80	1:40	-	-	-	-	-

-, not detected
